# Supplementary material for: Predicting visual function by interpreting a neuronal wiring diagram
Source: Nature. 2024 Oct 2;634(8032):113–23. doi: 10.1038/s41586-024-07953-5 (PMC11446822; doi:10.1038/s41586-024-07953-5)
Supplement: Supplementary file 6 — CRF and ERF predictions for individual TmY4 and TmY9 cells. Analogous to Supplementary Data 3, but for TmY target types. Shown are the top four monosynaptic pathways, the strongest pathway passing through each of the top ten intermediary types (ranking from Extended Data Fig. 7), and the trisynaptic pathway Tm1–TmY–Dm3–TmY (see the section entitled Prediction of spatial normalization). [file 41586_2024_7953_MOESM6_ESM.zip › DataS4/TmY9qΓèÑ/720575940643531168.pdf]

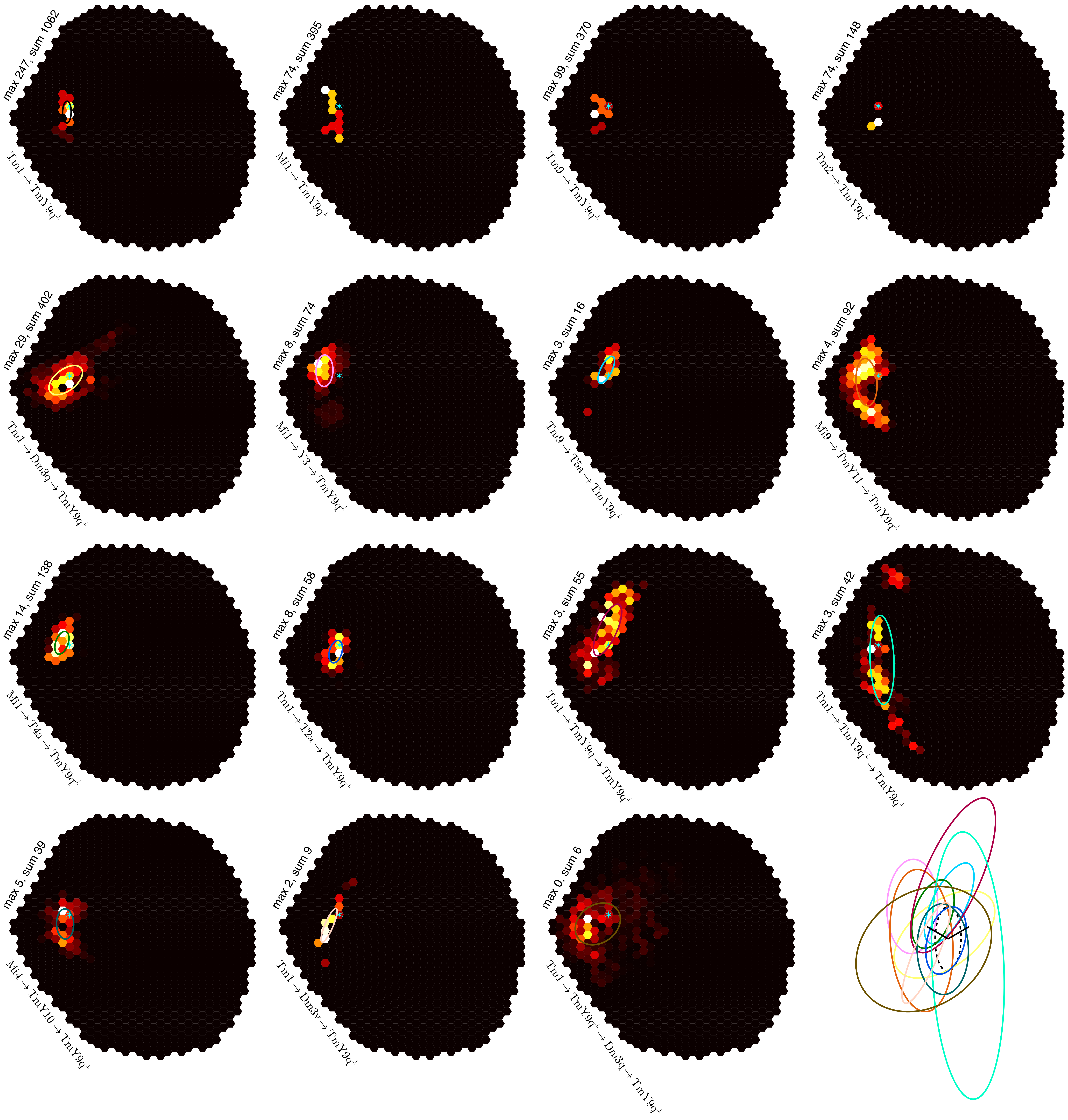
max 247, sum 1062
Tm1 → TmY9q⊥
max 74, sum 395
Mi1 → TmY9q⊥
max 99, sum 370
Tm9 → TmY9q⊥
max 74, sum 148
Tm2 → TmY9q⊥
max 29, sum 402
Tm1 → Dm3q → TmY9q⊥
max 8, sum 74
Mi1 → Y3 → TmY9q⊥
max 3, sum 16
Tm9 → T5a → TmY9q⊥
max 4, sum 92
Mi9 → TmY11 → TmY9q⊥
max 14, sum 138
Mi1 → T4a → TmY9q⊥
max 8, sum 58
Tm1 → T2a → TmY9q⊥
max 3, sum 55
Tm1 → TmY9q → TmY9q⊥
max 3, sum 42
Tm1 → TmY9q⊥ → TmY9q⊥
max 5, sum 39
Mi4 → TmY10 → TmY9q⊥
max 2, sum 9
Tm1 → Dm3v → TmY9q⊥
max 0, sum 6
Tm1 → TmY9q⊥ → Dm3q → TmY9q⊥
